# Identification of a Novel Gene Product That Promotes Survival of *Mycobacterium smegmatis* in Macrophages

**DOI:** 10.1371/journal.pone.0031788

**Published:** 2012-02-21

**Authors:** Assunta Pelosi, Danielle Smith, Rajini Brammananth, Agnieszka Topolska, Helen Billman-Jacobe, Phillip Nagley, Paul K. Crellin, Ross L. Coppel

**Affiliations:** 1 Department of Microbiology, Monash University, Clayton, Victoria, Australia; 2 Department of Biochemistry and Molecular Biology, Monash University, Clayton, Victoria, Australia; 3 Department of Microbiology and Immunology, University of Melbourne, Melbourne, Victoria, Australia; 4 Australian Research Council Centre of Excellence in Structural and Functional Microbial Genomics, Monash University, Clayton, Victoria, Australia; University of Maryland, United States of America

## Abstract

**Background:**

Bacteria of the suborder Corynebacterineae include significant human pathogens such as *Mycobacterium tuberculosis* and *M. leprae*. Drug resistance in mycobacteria is increasingly common making identification of new antimicrobials a priority. Mycobacteria replicate intracellularly, most commonly within the phagosomes of macrophages, and bacterial proteins essential for intracellular survival and persistence are particularly attractive targets for intervention with new generations of anti-mycobacterial drugs.

**Methodology/Principal Findings:**

We have identified a novel gene that, when inactivated, leads to accelerated death of *M. smegmatis* within a macrophage cell line in the first eight hours following infection. Complementation of the mutant with an intact copy of the gene restored survival to near wild type levels. Gene disruption did not affect growth compared to wild type *M. smegmatis* in axenic culture or in the presence of low pH or reactive oxygen intermediates, suggesting the growth defect is not related to increased susceptibility to these stresses. The disrupted gene, *MSMEG_5817*, is conserved in all mycobacteria for which genome sequence information is available, and designated *Rv0807* in *M. tuberculosis*. Although homology searches suggest that MSMEG_5817 is similar to the serine:pyruvate aminotransferase of *Brevibacterium linens* suggesting a possible role in glyoxylate metabolism, enzymatic assays comparing activity in wild type and mutant strains demonstrated no differences in the capacity to metabolize glyoxylate.

**Conclusions/Significance:**

*MSMEG_5817* is a previously uncharacterized gene that facilitates intracellular survival of mycobacteria. Interference with the function of MSMEG_5817 may provide a novel therapeutic approach for control of mycobacterial pathogens by assisting the host immune system in clearance of persistent intracellular bacteria.

## Introduction

Macrophages are major cellular components of innate host defence pathways and have roles including the recognition, ingestion and destruction of foreign microbes including pathogenic and non-pathogenic Mycobacteria. Pathogenic members of the genus have developed numerous strategies to evade the antimicrobial actions of the macrophage and to survive within this normally inhospitable cell eventually resulting in disease [1]. The interactions between the intracellular mycobacterial pathogens, such as *Mycobacterium tuberculosis*, and host are central to their success and have major implications for establishment of disease, spread of infection and persistence within the host. A characteristic of many mycobacterial diseases, particularly tuberculosis (TB), is that the infective pathogen can persist in the host for long periods of time in either a latent state where bacterial growth is stationary, or in a metabolically active state that facilitates proliferation. The World Health Organisation (www.who.int/tb/en) has estimated that one-third of the world's population have a latent TB infection that could potentially be activated given the correct conditions in an immunocompromised host.

Upon infection, most non-pathogenic microbes are internalised by host macrophages into a phagosome. Within the phagosome, the invading microbe is exposed to elevated levels of reactive oxygen intermediates (ROI) and reactive nitrogen intermediates in an increasingly hostile environment [2], [3], [4]. The phagosome matures and fuses with organelles of the endocytic pathway acquiring molecular markers that act to acidify the phagocytic compartment to pH 5, and hydrolytic enzymes that digest its contents [1]. In contrast, the intracellular mycobacterial pathogen is able to survive for long periods within the phagosome, creating a hospitable environment by modulating host-signalling pathways to alter vesicular membrane trafficking and phagolysosome formation. The resulting phagocytic compartments fail to mature, do not fuse with late endosomes and lysosomes and do not acquire lysosomal hydrolases [5], [6]. Moreover, mycobacterial phagosomes do not acidify below pH 6–6.5 due to a depletion of vesicular proton-ATPase (V-ATPase) at the phagosomal membrane [7], [8]). It is widely accepted that mycobacterial cell wall lipids, phosphatidylinositol mannoside (PIM) and lipoarabinomanann (LAM) play a major role in blocking phagosomal maturation [9]. LAM is thought to block vesicular trafficking thus inhibiting the acquisition of late endosomal and lysosomal cargo from the trans-Golgi network. Conversely, PIM is believed to promote fusion to early endosomes perhaps to supply nutrients to the phagosome and maintain a less acidic environment [6], [10]. The mycobacterial genes that mediate these adaptive responses are largely unknown.

In addition to internalization, recognition of mycobacteria by the host immune system, particularly by members of the Toll-like receptor (TLR) family of molecular pattern recognition receptors, is a crucial step for an effective host response. TLR recognition of mycobacteria or mycobacterial products is particularly important for the production of the pro- and anti-inflammatory cytokines and chemokines responsible for the progression or containment of infection.

Identification of the mechanisms used by mycobacteria to subvert the antibacterial properties of the macrophage is important for understanding mycobacterial virulence and disease. The *mce1* operon, originally identified in *M. tuberculosis* [11] but also present in other pathogenic and non-pathogenic mycobacteria [12], has been shown to be important in the invasion of the mammalian host cell and the establishment a persistent infection in the mouse model [11], [13], [14]. A number of genes from *M. tuberculosis* including a catalase peroxidase, *katG* and alkylhydroperoxide reductase, *ahpC* have suggested functions in conferring resistance to ROI [15], [16]. Other genes have been implicated in modulating phagosomal maturation and promoting intracellular survival including, mycobacterial protein kinase G (*pknG*) from pathogenic mycobacteria [17] and a hypothetical gene from *M. marinum*, *pmiA* [18]. Also implicated in intracellular mycobacterial survival are *icl* (isocitate lyase), *mptpB* (tyrosine phosphatase) and *sapM* (PI3P phosphatase) from *M. tuberculosis* [19], [20], [21].

Previous studies have shown that while *M. smegmatis* is generally considered non-pathogenic, it does have a limited capacity to survive and multiply within macrophages and delay phagosomal acidification making it a suitable model system to study intracellular mycobacterial survival [8] [22]. Here, we describe the intracellular phenotype of *M. smegmatis* mutants with insertions in a highly conserved hypothetical gene (*MSMEG_5817*). The intracellular survival kinetics of the mutants suggest that MSMEG_5817 functions in mycobacterial survival in host macrophages.

## Results

### Identification of the transposon insertion site in Myco132 and analysis of flanking sequences

In previous studies we screened members of a random Tn*611* transposon mutant library of *M. smegmatis* for a number of different phenotypes [23], [24], [25]. Myco132 was originally isolated from this library based on its altered colony morphology and capacity to take up dyes from the growth media. To identify the gene disrupted by the Tn*611* element in Myco132, genomic DNA fragments flanking the transposon insertion site were obtained by ligation-mediated polymerase chain reaction (LMPCR) [26] and sequenced. The sequences obtained were assembled and searched against the *M. smegmatis* genome (http://cmr.jcvi.org/cgi-bin/CMR/CmrHomePage.cgi) using the BLAST algorithm. The gene containing the transposon insertion was identified as *MSMEG_5817*, encoding a conserved hypothetical protein of 128 amino acid residues with orthologs in a number of pathogenic mycobacteria (Table 1).

**Table 1:** Percent amino acid identity and similarity between MSMEG_5817 orthologs.

	*M. smegmatis*	*M. tuberculosis*	*M. paratuberculosis*	*M. avium*	*M. leprae*
*M. smegmatis*	100	72	70	69	72
*M. tuberculosis*	66	100	73	74	80
*M. paratuberculosis*	65	71	100	94	73
*M. avium*	63	70	93	100	77
*M. leprae*	62	73	65	70	100

Shaded and unshaded areas indicate % similarity and % identity, respectively.

**Figure 1:**
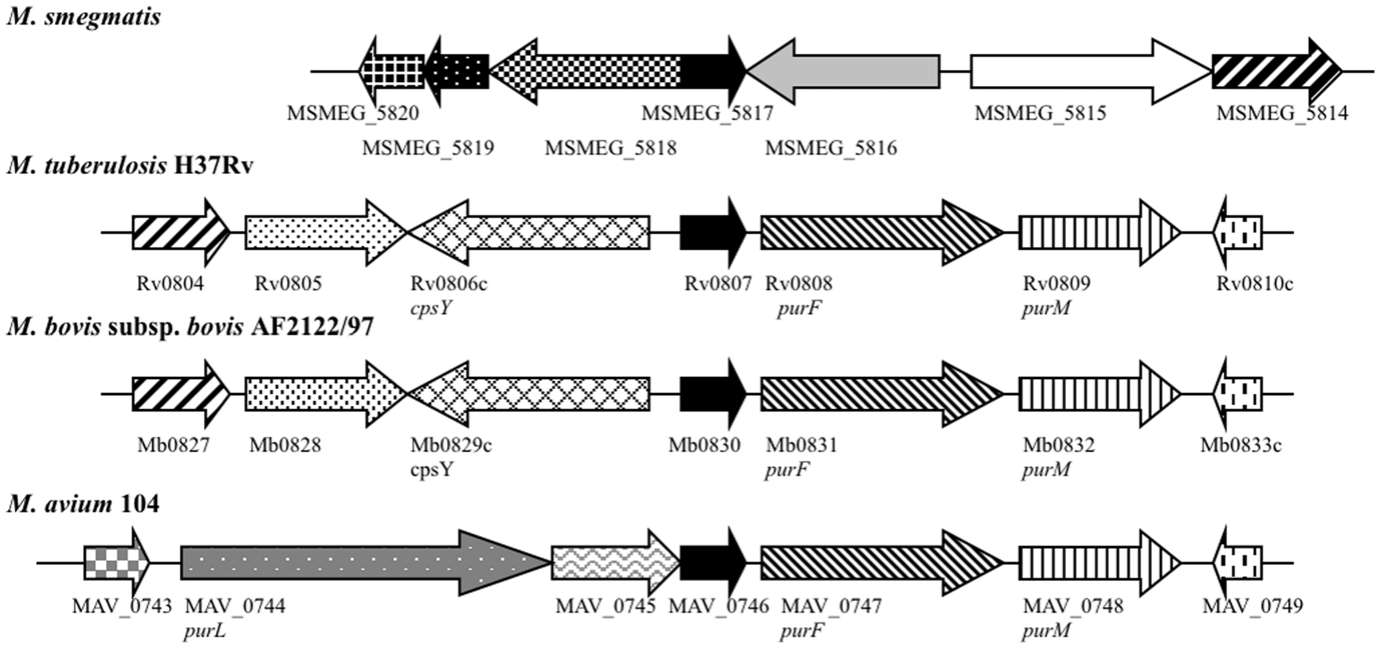
Schematic representation of the gene arrangement in the region of the *M. smegmatis MSMEG_5817* locus and orthologous regions in *M. tuberculosis* H37Rv, *M. bovis* subsp. *bovis* AF2122/97 and *M. avium* 104. Arrows indicate the relative direction of transcription. Shading indicates homologous genes.

**Figure 2:**
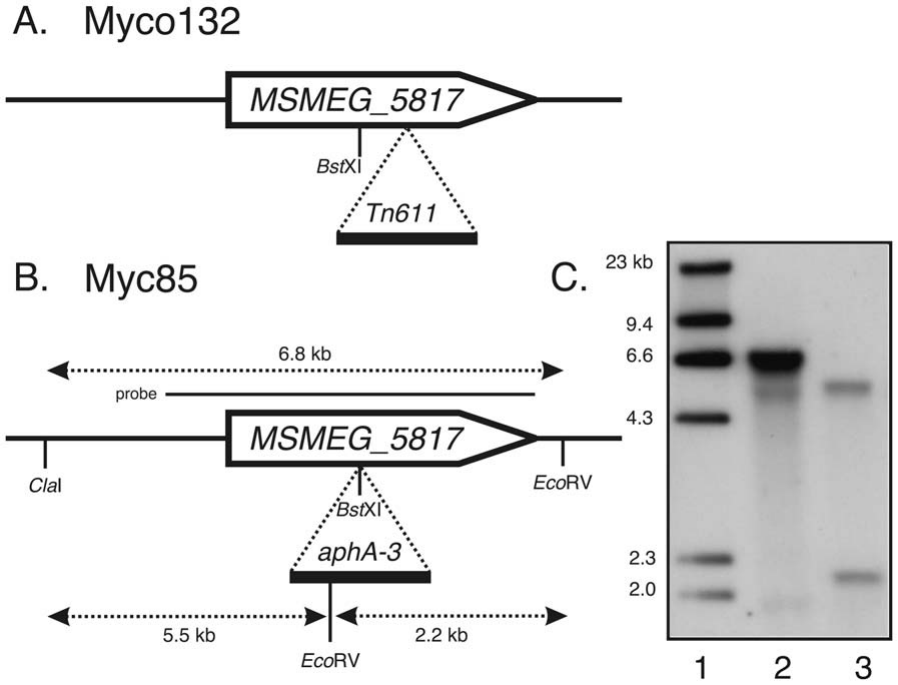
Transposon Tn*611* and targeted inactivation of the *MSMEG_5817* gene. (A) Insertion site of the Tn*611* element in Myco132. (B) Targeted disruption of *MSMEG_5817* by a kanamycin resistance gene in strain Myc85. The insertion of the *aphA-3* gene at the unique *Bst*XI site in *MSMEG_5817* is indicated. Position of the probe used for Southern hybridization is shown as a solid horizontal line. Restriction fragments detected by the probe in WT and Myc85 are shown as broken horizontal lines. (C) Southern hybridization analysis of Myc85. Lane 1, molecular weight markers with sizes indicated in kilobases (kb); Lane 2, wild-type *M. smegmatis* genomic DNA digested with *Cla*I/*Eco*RV; Lane 3, Myc85 genomic DNA digested with *Cla*I/*Eco*RV.

Sequence analysis of the 5′ flanking region revealed a second gene, immediately upstream of the disrupted *MSMEG_5817* gene (Fig. 1). This second gene, identified as *MSMEG_5818* encodes a putative virulence factor mammalian cell entry (Mce) family protein, sharing significant homology at the amino acid level to *mce1*A (68%) but is not located within the *mce1* operon in *M. smegmatis*. In addition to the defining *mce* domain, *MSMEG_5818* also contains a putative inner membrane component binding-protein-dependent transport system. Upstream of this gene is *MSMEG_5819* encoding a putative pyridoxamine 5-phosphate oxidase and a hypothetical protein-encoding gene, *MSMEG_5820*. The sequence flanking the 3′ end of *MSMEG_5817* corresponds to *MSMEG_5816*, encoding a different conserved hypothetical protein. Searches for amino acid homology did not reveal a *MSMEG_5816* ortholog in *M. tuberculosis*; instead the closest significant match was to a conserved hypothetical protein, nfa21710 from *Nocardia farcinica* (70%; *E* = 1e^-130^). The next two 3′ genes are *MSMEG_5815* and *MSMEG_5814* encoding betaine aldehyde dehydrogenase (EC 1.2.1.8) and 4-carboxymuconolactone decarboxylase domain protein (EC 4.1.1.44) respectively. In each case, the three ORFs identified flanking each side of *MSMEG_5817* are transcribed in the opposite direction to *MSMEG_5817*. Interestingly, the gene arrangement in the region defined by these seven ORFs identified in *M. smegmatis* diverges from that found in *M. tuberculosis*, *M. bovis* subsp. *bovis* and *M. avium*, where arrangement of the respective *MSMEG_5817* orthologs and 3′ flanking the genes is conserved (Fig. 1). Closer inspection of the sequences flanking the transposon insertion identified the insertion site as nucleotide 222 of the 387 bp gene (Fig. 2a).

### Myco132 has a defect in early intracellular survival

Preliminary studies using defective survival in J774A.1 macrophages as the readout indicated that Myco132 was unable to proliferate over a 4 h infection period, unlike several other mutants selected from the transposon library. To better characterize the phenotype, intracellular survival of Myco132 in J774A.1 macrophages was compared to wild type *M. smegmatis* mc^2^155 (WT) over a 48 h infection period. Following infection, macrophages were incubated for 1 h to permit phagocytosis, after which the non-phagocytosed bacteria were removed and the culture initiated. At each time point, intracellular mycobacteria were released from J774A.1 macrophages and the viable count determined by measuring colony-forming units (CFU) of bacteria resident within macrophages.

**Figure 3:**
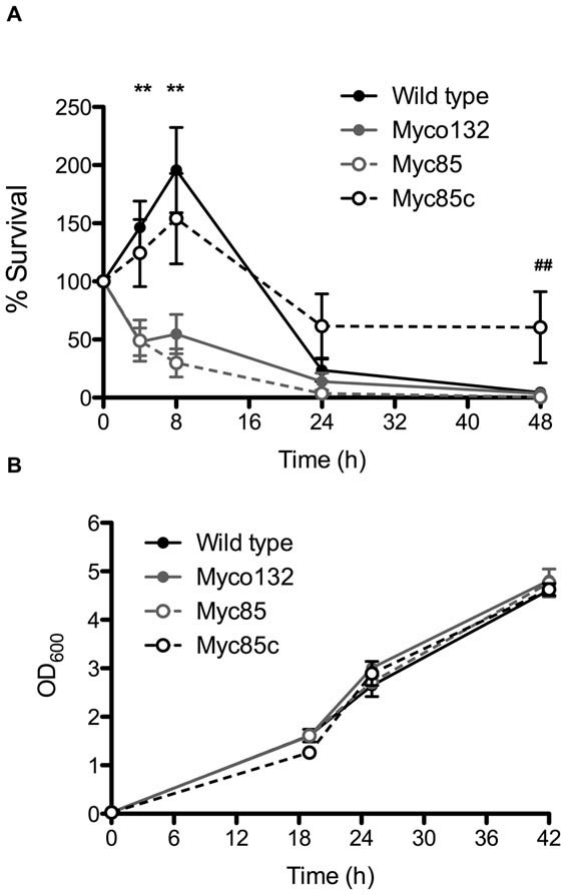
Growth of WT *M. smegmatis*, Myco132, Myc85 and Myc85c inside J774A.1 macrophages and *in vitro*. (A) Intracellular survival of WT, Myco132, Myc85 and Myc85c in J774A.1 macrophages over a 48 h infection (n = 3–19). Numbers of intracellular bacteria are shown as a percentage of the numbers detected at t = 0 h (% survival). Significant differences were determined by Student's t-test and are indicated by ** (p<0.05) between WT/Myc85c and Myco132/Myc85 and ## (p<0.05) between WT and Myc85c. Error bars represent SEM. (B) *In vitro* growth of WT, Myco132, Myc85 and Myc85c in BHI (n = 3). Error bars represent SD.

Myco132 exhibited a marked reduction in intracellular survival during the first 24 h of infection compared to WT (Fig. 3a). At 4 h infection Myco132 (48.0%±11.8) showed ∼3-fold decrease in viability compared to WT (146.4%±22.7). This difference was more pronounced at 8 h with the proportion of intracellular Myco132 (54.7%±16.8) bacteria recovered from J774A.1 macrophages still significantly lower than WT (195.7%±36.6). By 24 h all strains were equally sensitive to macrophage killing.

### Targeted disruption of *MSMEG_5817* and complementation

To confirm that the macrophage survival defect observed for Myco132 was due solely to the disruption of the *MSMEG_5817* gene, we constructed a new strain in which *MSMEG_5817* was disrupted by a kanamycin resistance gene (Fig. 2b) using a two-step recombination strategy (see Materials and Methods). Briefly, the *MSMEG_5817* gene was PCR amplified, cloned into pUC18 [27] and disrupted by insertion of a kanamycin-resistance gene at a unique *Bst*XI site. This fragment was subcloned into the temperature-sensitive vector pPR27 [28] followed by introduction into *M. smegmatis* and selection for single crossovers at the non-permissive temperature. Double crossovers were then derived by selection of plates containing kanamycin and sucrose. A potential *MSMEG_5817*:*aphA3* mutant was confirmed using Southern hybridization (Fig. 2c) and designated Myc85. To complement Myc85, the *MSMEG_5817* gene was PCR amplified and cloned into a modified version of the mycobacterial expression vector pMV261 [29] in which the kanamycin resistance gene had been replaced by a gentamycin resistance gene. This complementation plasmid was introduced into Myc85, creating Myc85c.

To determine the capacity of the targeted knockout and complementation strain to survive *in vivo*, the macrophage survival experiments described above for Myco132 were repeated using Myc85 and Myc85c (Fig. 3a). At 4 h infection Myc85 (49.1%±17.7) showed ∼3-fold decrease in viability compared to WT (146.4%±22.7). This difference was more pronounced at 8 h with the proportion of intracellular Myc85 (29.9%±12.0) bacteria recovered from J774A.1 macrophages still significantly lower than WT (195.7%±36.6). Thus the capacity of Myc85 to survive in J774A.1 macrophages was found to strongly resemble that of Myco132. In contrast, the early survival kinetics of Myc85c closely resembled those observed for WT, suggesting that intracellular survival had been restored to WT levels by expression of the *MSMEG_5817* gene product. Interestingly, Myc85c was still viable at 48 h post infection while WT, Myco132 and Myc85 had been cleared by this time point (Fig. 3a).

The observed failure of Myco132 and Myc85 to proliferate in macrophages could reflect a specific intracellular growth defect or a more general problem with replication. To determine this we examined their capacity to grow in axenic, liquid broth cultures. When cultured in BHI broth, WT, Myco132, Myc85 and Myc85c showed similar rates of growth over a 42 h period (Fig. 3b). Since Myco132 and Myc85 behaved almost identically *in vitro* and *in vivo*, we selected Myco132 as the representative *MSMEG_5817* mutant for all subsequent analyses.

**Figure 4:**
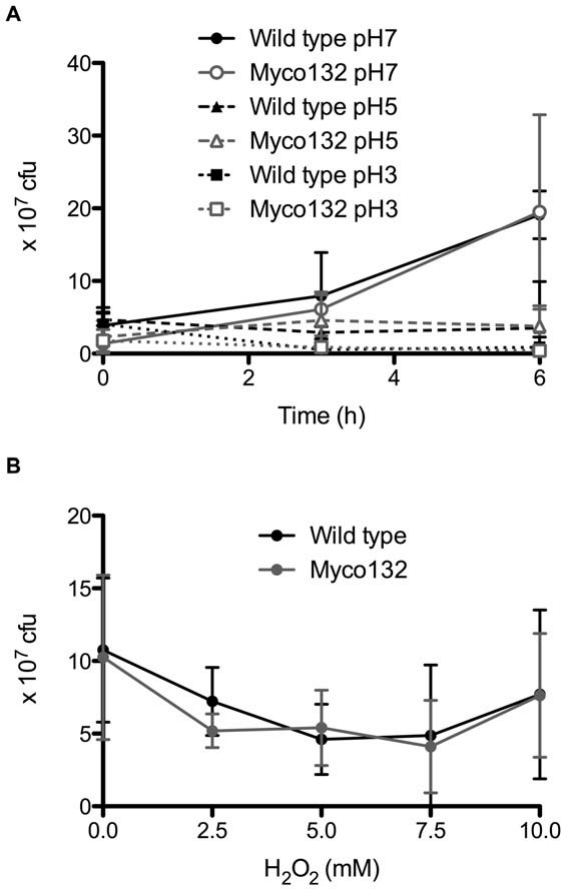
Growth of WT *M. smegmatis* and Myco132 under stress conditions. (A) *In vitro* growth of WT and Myco132 at different pH (n = 3–4). Error bars represent SD. (B) *In vitro* growth of WT and Myco132 at pH 5 in the presence of H_2_O_2_ for 6 h (n = 3–4). Error bars represent SD.

To determine whether the observed reduction in intracellular survival of Myco132 could be accounted for by antimicrobial mechanisms of macrophages, the sensitivity of WT and Myco132 strains to low pH or the presence of ROI was examined *in vitro* over a 6 h growth period. The growth rate in acidic conditions was compared in BHI broth over a pH range that included pH 7, pH 5 and pH 3 (Fig. 4a). Similarly, to assess the impact of ROI, H_2_O_2_ was added to BHI (pH 5) at various concentrations (Fig. 4b). In each case growth of Myco132 did not differ significantly from WT in response to either acid or H_2_O_2_ stress.

To assess acid sensitivity in more detail, Myco132 was also compared to WT for differences in intracellular pH regulation, sensitivity to protonophores at low pH and acid stress survival. Firstly, the regulation of pH homeostatis by *M. smegmatis* was studied by determining the intracellular pH as a function of external pH. Internal pH was measured by [^14^C] benzoic acid equilibration in cells oxidising glucose but there were no significant differences observed between WT and Myco132 (data not shown). Secondly, acid sensitive strains should be unable to grow in 7H9 medium (pH 5.0) in the presence of 5 µM carbonyl cyanide m-chlorophenylhydrazone (CCCP), a concentration that is not inhibitory to the WT. However, Myco132 showed comparable growth to WT at pH 5.0 at 1 to 8 µM CCCP. Finally, cells were grown at pH 5.0 and then exposed to a lethal pH 2.7 to test acid stress survival. WT cultures showed a decline in viability of 23% in the first 4 hr at pH 2.7 and an 80% decline after 6 hours. Myco132 viability declined by 42% within 4 hours and was not considered significantly different from WT. Collectively, our data suggest that the failure of Myco132 to survive intracellularly was not due to increased sensitivity to conditions encountered within macrophages.

### Phagocytosis of Myco132 is not reduced compared to wild type bacteria

**Figure 5:**
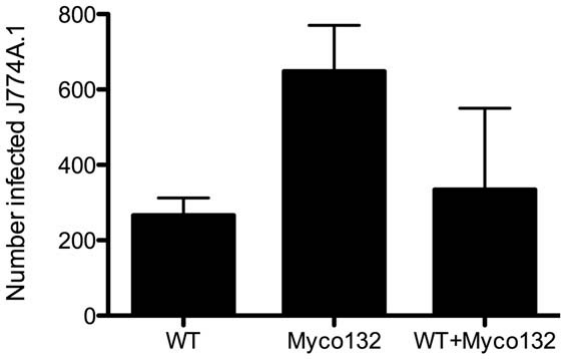
The mean number of J774A.1 cells infected with SYTO9 or SYTO62 stained WT and Myco132. Infected macrophage cells with phagosomes harbouring either WT or Myco132 alone, or both *M. smegmatis* strains simultaneously was determined from 11,000–15,000 J774A.1 cells from two experiments by FACS. Error bars represent SD.

To determine whether the reduced number of intracellular Myco132 observed was due to a defect in the attachment or uptake of the mutant bacteria by macrophages, WT and Myco132 bacteria were fluorescently stained with SYTO9 and SYTO62, respectively, and presented to macrophage cells simultaneously. FACS was then used to detect macrophages infected with one or both strains. Dye swaps were also performed to ensure the fluorescent label used did not differentially influence phagocytosis. The analysis revealed that phagocytosis of Myco132 was not deficient. Whilst the number of macrophages infected with Myco132 alone was at least double that observed for WT (Fig. 5), this was not statistically significant. Similarly when macrophages were presented with both strains simultaneously, phagocytosis was not biased towards either WT or mutant, suggesting that the mutant is not defective in attachment or uptake but rather in its ability to survive once internalised by the macrophage.

### Myco132 causes increased NF-κB activation in macrophage cells

**Figure 6:**
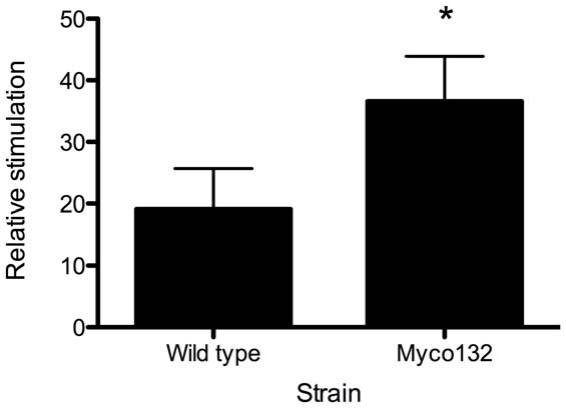
NF-κB activation in macrophages infected with WT *M. smegmatis* or Myco132. RAW264.7 mouse macrophage cells stably expressing the NF-κB-dependent ELAM-luciferase reporter construct were exposed to 10^5^ cfu formaldehyde-killed wild-type *M. smegmatis* or Myco132 for 3.5 hours. Mean relative stimulation of luciferase activity for three biological replicates per strain ± SD for a representative experiment from 3 experiments, each performed in triplicate, is shown. Significant differences were determined by Student's t-test and are indicated by * (*p*<0.05).

Although phagocytosed as well or more than WT *M. smegmatis*, Myco132 showed a marked defect in early cellular survival. Besides phagocytosis, recognition of mycobacteria by TLRs can induce several signalling pathways that converge at the level of NF-κB activation and mediate cell activation and cytokine production. Therefore, using an NF-κB-dependant reporter gene system as a measure of TLR-dependant activation, we investigated whether Myco132 was able to activate NF-κB to a similar level as WT *M. smegmatis*. Whereas both *M. smegmatis* and Myco132 induced a significant increase in NF-κB activation above unstimulated cells (Fig. 6), there was a statistically significant increase in NF-κB activation demonstrated with Myco132 compared to a WT *M. smegmatis* control.

### Similarity searches of *MSMEG_5817*

**Figure 7:**
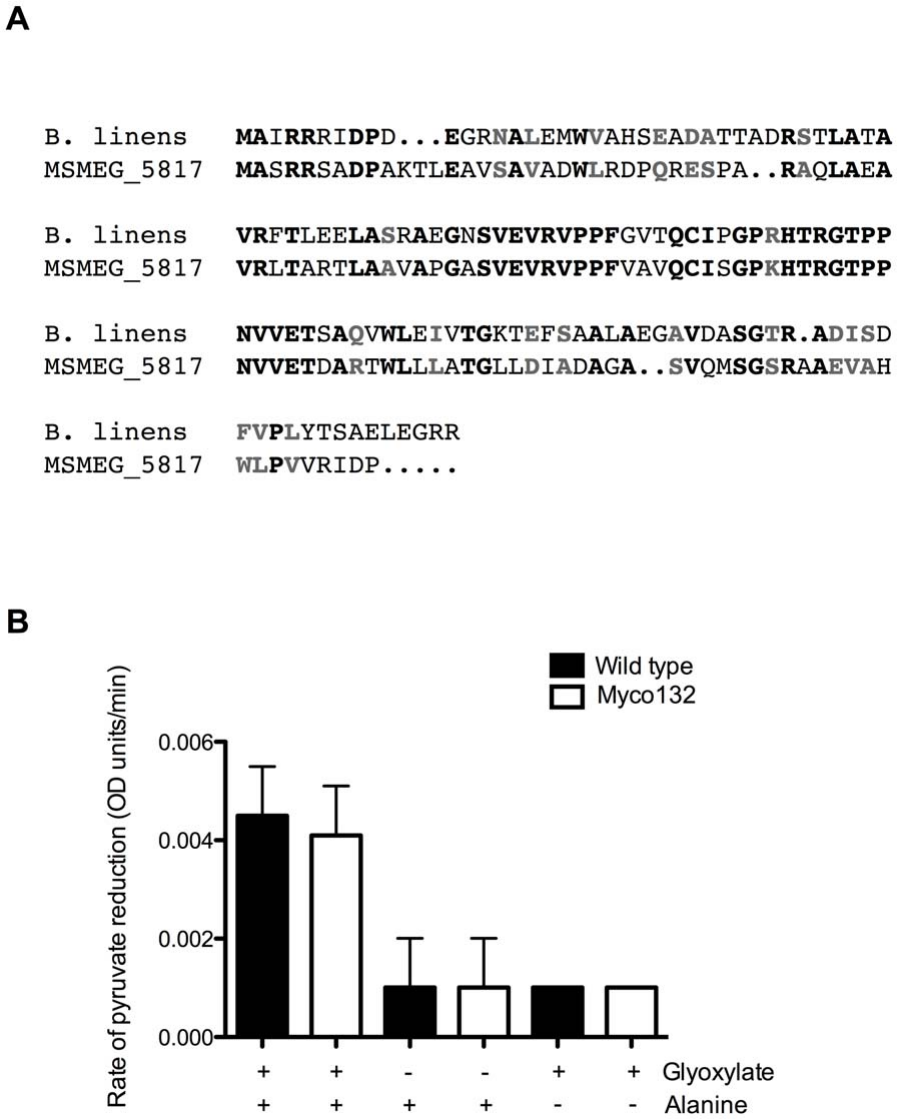
A. ClustalW amino acid sequence alignment of MSMEG_5817 and *B. linens* SPT/AGT (accession NZ_AAGP01000002). Identical amino acid residues are denoted by **black** type, conserved residues by **grey**. **B. The rate of pyruvate reduction in WT and Myco132** ***M. smegmatis*** **strains (n = 4).** Negative controls were performed in the absence of glyoxylate or alanine.

BLASTP searches of the non-redundant protein sequence (nr) database (http://www.ncbi.nlm.nih.gov/BLAST/) were performed in an attempt to identify other proteins with homology to MSMEG_5817 to gain insight into its possible function. These searches yielded 37 significant matches, 36 of which were to unknown hypothetical proteins from various species of *Mycobacterium*, *Rhodococcus* and *Corynebacterium*. Significant homology (62% similarity and 46% identity; *E* = 6e-15) was also detected to serine:pyruvate aminotransferase (SPT), also known as alanine:glyoxylate aminotransferase (AGT) from *Brevibacterium linens* BL2 (accession ZP_00381195) (Fig. 7a). Comparison of SPT/AGTs from a number of organisms revealed a conserved aminotransferase class V domain. This domain is absent in both MSMEG_5817 and the *B. linens* predicted SPT/AGT.

### *MSMEG_5817* is transcribed in *M. smegmatis* and encodes a protein of unknown function

The possibility that *MSMEG_5817* encodes a functional AGT, mediating transfer of the amino group of alanine to glyoxylate to form glycine and pyruvate was investigated. To test this, alanine and glyoxylate were added to enzyme extracts prepared from WT and Myco132 lysates. Pyruvate formed by AGT was then measured by spectrophotometric NADH determination using LDH. To ensure the enzyme preparation did not contain other substrates that would permit formation of pyruvate, the reaction was also performed in the absence of glyoxylate or alanine. The relative rate of pyruvate reduction determined by the extinction of NADH in the subsequent LDH assay was very similar for WT and Myco132 suggesting that AGT activity in the two strains was not noticeably different (Fig. 7b). We concluded that *MSMEG_5817* does not encode a functional AGT under the conditions tested.

**Figure 8:**
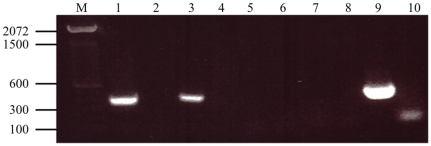
RT-PCR analysis of wild-type *M. smegmatis* and Myco132 mRNA. mRNA was isolated from intracellular and extracellular bacteria and amplified by RT-PCR. Products were resolved on a 1% agarose gel. Lane M, TrackIt™ 100 bp DNA ladder (Invitrogen). Lanes 1, 3, 5, 7: intracellular WT, extracellular WT, intracellular Myco132 and extracellular Myco132, respectively. Lanes 2, 4, 6, 8: RT minus controls for intracellular WT, extracellular WT, intracellular Myco13 and extracellular Myco132, respectively. Lane 9: gDNA positve control. Lane 10: negative PCR control.

Finally, RT-PCR was performed to detect *MSMEG_5817* mRNA in intra- and extracellular samples of *M. smegmatis* WT and Myco132 strains. In WT, amplification was achieved from both the intra- and extracellular samples (Fig. 8). Absence of a PCR product in the RT minus controls confirmed amplification was achieved from cDNA and not gDNA. Amplification was not detected in either the intra- or extracellular Myco132 samples suggesting that disruption of *MSMEG_5817* in the mutant abolished transcription of this gene.

## Discussion

In this study we have identified a novel gene from *M. smegmatis* involved in intracellular survival in host macrophages. Although *M. smegmatis* is considered non-pathogenic, it has been shown to behave in a pathogen-like manner by manipulating the host cell during the initial stages of infection to delay acidification and recruitment of V-ATPase to the phagosome [8], [22]. *M. smegmatis* has been used as a model species in previous studies to successfully identify genetic loci implicated in intracellular survival [26]. Here we have used the J774A.1 macrophage cell line derived from a tumour of a BALB/c adult female mouse for our infection model. J774A.1 macrophages have been used widely to study intracellular mycobacterial survival and host interactions [6], [8], [18], [20], [22]. We report that *M. smegmatis* mutant Myco132 carries a transposon inserted into *MSMEG_5817*, a gene encoding a hypothetical protein of unknown function. This mutant exhibits reduced intracellular survival in J774A.1 macrophages. This defect in growth is specific to the phagosomal compartment and is not observed *in vitro* broth culture. Furthermore, this defect cannot be attributed to an increased sensitivity of Myco132 to acidic pH or the presence of ROI.

The survival kinetics observed for WT *M. smegmatis* were in general agreement with the three rapid killing phases described by Anes *et al* over a 48 h infection period [22]. Myco132 and Myc85 were more sensitive to the antimicrobial macrophage environment than WT during the initial phase of killing with significantly fewer intracellular bacteria surviving beyond 4 h. In fact, while WT undergoes an intracellular growth phase between killing phases 1 and 2, Myco132 does not recover as intracellular survival continues to decline. During the second phase of killing, WT and mutant strains were almost completely eliminated until finally disappearing during the third killing phase when the macrophage achieves complete clearance at 48 h. Complementation of the mutant with a plasmid-encoded copy of the *MSMEG_5817* gene restored early survival capacity to Myc85, but also produced a strain that persisted longer than either WT or the mutant strains. Since multiple plasmid-encoded copies of *MSMEG_5817* are likely to result in overexpression of the gene, this finding could indicate a role for MSMEG_5817 in persistence due to overproduction of MSMEG_5817 in the complemented strain.

*MSMEG_5817* was identified as the gene disrupted by the transposon insertion and has orthologs in both pathogenic and non-pathogenic mycobacteria. This is consistent with the evolving view of pathogenicity based on genomic analysis that has shown that many virulence factors are present in both virulent and non-virulent species, presumably as a consequence of similarity in selective forces under differing environmental conditions [30]. In *M. tuberculosis*, the *MSMEG_5817* ortholog *Rv0807* was not detected as being essential for survival in macrophages in a broad genomic screen using a transposon site hybridisation (TraSH) approach [31]. However, examination of the data suggests that the small size of the gene may have precluded it from the analysis due to a lack of *mariner* insertions, highlighting a possible limitation of the TraSH approach. *Rv0807* is also not essential for growth of *M. tuberculosis* [32], suggesting that MSMEG_5817 may contribute to intracellular survival through a mechanism common to pathogenic and non-pathogenic species, perhaps to perform basic cellular functions that are not exclusive to promoting bacterial survival within host macrophages. This may explain why Myco132 growth is normal in broth culture yet defective in the macrophage. This is not unusual, given that the glyoxylate shunt enzyme encoded by *icl*, is also not essential in *M. tuberculosis*, yet required for intracellular survival [19] [32]. Like Myco132, impaired growth of *icl* deficient *M. tuberculosis* was observed exclusively within macrophages [19]. Transcription of *MSMEG_5817* was detected in both intracellular and extracellular WT *M. smegmatis* suggesting that it is not simply the presence of MSMEG_5817 transcript that is important in promoting intracellular survival but perhaps its relative abundance in response to the hostile macrophage environment. A quantitative analysis of transcript levels is warranted to determine whether *MSMEG_5817* is differentially regulated during the different phases of macrophage infection.

NF-κB activity co-ordinates the immune response for resistance to a wide range of pathogens. Activation of NF-κB by mycobacterial components (lipoproteins, Ara-LAM, lipomannan and Man-LAM [33]) has been shown to be critical for clearance by the host. It is possible that in addition to maintaining basic bacterial functions, the protein encoded by *MSMEG_5817* may modulate NF-κB activity in infected macrophages in an, as yet, unidentified manner. Certainly, other pathogenic bacterial products are known to suppress NF-κB signalling pathways to promote their survival. For example, by decreasing NF-κB activity, uropathogenic *Escherichia coli* and non-pathogenic *Salmonella* promote internalisation by host cells, inducing minimal inflammation and subsequent host immune responses compared to other pathogens [34]–[35]. Perhaps more relevant to our results, the toxic *Mycobacterium ulcerans* product mycolactone (critical for *M. ulcerans* pathology *in vivo* but not *in vitro* bacterial growth) has been shown to inhibit NF-κB activity in a wide variety of infected cells [36].

For many bacteria, the activation of NF-κB is in large part due to the interaction of different bacterial components with host TLRs and these interactions have a significant effect on the establishment of infection. Macrophage responses upon TLR stimulation relevant to anti-mycobacterial responses include upregulation of bacterial phagocytosis, phagosome maturation, expression of pro-inflammatory cytokines, RNI-mediated microbicidal mechanisms and production of cathelicidin, an antimicrobial peptide. Thus, it is possible the hypothetical protein encoded by *MSMEG_5817* suppresses or blocks TLR-mediated NF-κB activity to promote survival of wild type *M. smegmatis in vivo*.

It is interesting to note that in *M. smegmatis*, a *mce1A* paralog is found immediately upstream of *MSMEG_5817*. In *M. tuberculosis*, *mce1A* is located in the *mce1* operon comprising eight genes [37]. An orthologous operon has also been identified in *M. smegmatis* [38] however *MSMEG_5818* is not located within this operon, suggesting that *mce1A* may have undergone duplication in this species. The *mce* locus was first implicated in host cell invasion and intracellular survival when Arruda et al. showed that expression of a fragment of *M. tuberculosis* DNA, conferred the ability of a non-pathogenic strain of *E. coli* to enter and survive within HeLa and macrophage cells [11]. Later studies showed that when this locus was inactivated in *M. bovis* BCG, the *mce1* mutant strain was reduced in its ability to enter HeLa cells [13]. A role in establishing a stable persistent infection was later reported when disruption of the *mce1* operon in *M. tuberculosis* resulted in a hypervirulent mutant strain that replicated continuously to kill infected mice more rapidly than WT *M. tuberculosis* [14]. The presence of putative transport domains in *mce* operons suggests an additional role for the operons in active transport across the membrane, possibly in response to environmental signals such as nutrient requirement and stress [38]. Kumar et al speculate that this may be the primary role of Mce family proteins in less virulent mycobacteria such as *M. smegmatis* [38]. Given that a putative transport domain was identified in MSMEG_5818, a similar function in transport should not be discounted.

We can be certain that the phenotype observed in Myco132 and Myc85 is a consequence of disruption of *MSMEG_5817* because complementation with this gene alone prevents early destruction in the macrophage. Additionally, the tail to head arrangement of the two genes makes it unlikely that gene targeting of *MSMEG_5817* would have had any effect on transcription of this *mce* paralog. Whether this region is part of a genomic region that is involved in intracellular survival is unclear. The arrangement of genes rules out a simple operon arrangement and the significance of the juxtaposition of these two genes, if any, is unclear given that this gene arrangement is not conserved in *M. tuberculosis*.

A possible role for MSMEG_5817 as a SPT/AGT in glyoxylate metabolism was suggested on the basis of conserved amino acid homology to SPT/AGT from *B. linens*. We were unable to measure any detectable loss of enzymatic activity in the knockout line compared to WT *M. smegmatis* even though MSMEG_5817 was shown to be transcribed in the WT strain from our RT-PCR experiments. This finding led us to re-examine the sequence similarity studies. AGT (EC 2.6.1.44) and SPT (EC 2.6.1.51) have been identified in a variety of organisms including yeast, Arabidopsis and human where all examples have either a conserved aminotransferase class-III or V domain which is not present in either MSMEG_5817 nor the *B. linens* predicted SPT/AGT protein. In fact no patterns, motifs or domains were predicted for either sequence using the tools and software available on the ExPASy (Expert Protein Analysis System) proteomics server of the Swiss Institute of Bioinformatics (SIB). Moreover, sequence homology searches between characterised SPT/AGTs from eukaryotes and prokaryotes revealed no obvious ortholog in *M. smegmatis*, *M. tuberculosis*, *M. avium* and *M. bovis*. We conclude that this homology is an annotation error that should be removed from the sequence databases.

AGT functions in the detoxification of glyoxylate, an intermediate already implicated in latency in mycobacteria. Although common amongst eukaryotes, AGT has been identified in few prokaryotes, including *Rhodopseudomonas acidophila* [39] and *Thermococcus litoralis* [40]. Although not encoded by MSMEG_5817, specific AGT enzyme activity was detected in *M. smegmatis* lysates suggesting another protein is responsible that cannot be predicted by sequence similarity. Likely candidates are PLP-dependent aminotransferase class-III (AGT2) and class-V (AGT1) proteins of which there are numerous examples in *M. smegmatis* including MSMEG_0277, MSMEG_0782, MSMEG_5211 (class-III) and MSMEG_0978, MSMEG_6246 and MSMEG_6591.

In summary, we found that disruption of *MSMEG_5817* from *M. smegmatis* by transposon insertion resulted in failure to resist macrophage killing, leading to impaired survival in infected macrophages. However, the implications of this finding in pathogenic mycobacteria still remain unclear. Further investigation is warranted to determine the precise function of the encoded protein during infection. Transcriptome, proteome or metabolome-based approaches may be more fruitful in identifying related genes or metabolic pathways that may be influenced by the actions of this gene or gene product during infection of macrophages. Localization of the *MSMEG_5817* encoded product, particularly in pathogenic mycobacteria, may elucidate the host signalling pathway/s directly targeted during mycobacterial infection and disease progression. Although studies in *M. smegmatis* may not reveal genes specifically involved in virulence or pathogenesis, this species is a useful model to identify genes that are nonetheless important for intracellular survival and/or persistence during mycobacterial infections, and thus may present as attractive targets for new drug therapies.

## Materials and Methods

### *M. smegmatis* and macrophage strains and culture conditions

All bacterial strains were grown at 39°C in BHI broth supplemented with 0.05% Tween 80, on BHI agar plates, or in Middlebrook 7H9 broth with 10% DC enrichment (2% glucose, 0.85% NaCl). Transposon mutant Myco132 and targeted knockout Myc85 were cultured with the addition of 20 µg/ml kanamycin and the complementing strain Myc85c with 20 µg/ml kanamycin and 10 µg/ml gentamicin. Unless otherwise stated, all broths contained Tween 80 and all liquid and solid media were supplemented with the appropriate antibiotic. Murine macrophage cell line J774A.1 (ATCC TIB-67) was cultured in complete RPMI comprising RPMI 1640 (GIBCO) supplemented with 10% heat-inactivated foetal calf serum and 20 mM L-glutamine at 37°C, 5% CO_2_, in 75 cm^2^ vented T-flasks (Sarstedt).

### Isolation of *MSMEG_5817::Tn611* mutants and identification of transposon insertion sites

Myco132 was isolated from a library of *Tn611* insertion mutants of *M. smegmatis* [23]. The transposon insertion site was determined by the LMPCR method described by Prod'hom et al (1998) [26]. PCR products representing the genomic sequences flanking each side of the *Tn611* insertion site were sequenced using Big Dye Terminator reactions and resolved on an ABI 373A Automated Fluorescent Sequencer (Applied Biosystems Inc.).

### Construction and complementation of Myc85

*MSMEG_5817* and flanking sequences were PCR amplified using primers 5′-GCTCTAGACCCGCGCAGCAGGTAGGGGC-3′ and 5′-CGGGATCCGTTCGTGCCGAGGACCGCGAC-3′ and cloned into the *Xba*I and *Bam*HI (underlined) sites of pUC18 [27]. A non-polar kanamycin resistance cassette was inserted at the unique *Bst*XI site within *MSMEG_5817* and the entire *MSMEG_5817*::*aphA3* fragment subcloned into the temperature-sensitive vector pPR27 [28] using *Xba*I/*Bam*HI. This plasmid was introduced into *M. smegmatis* by electroporation at 30°C and single crossover strains were selected on kanamycin and gentamycin plates at 42°C. To derive a double crossover, one of these clones was grown in LB broth containing kanamycin then spread on LB plates containing kanamycin and 10% (w/v) sucrose. Genomic DNA was prepared from kanamycin resistant/gentamycin sensitive clones, digested with *Cla*I/*Eco*RV and subjected to Southern hybridization analysis with a *MSMEG_5817*-specific probe. To complement Myc85, the 387 bp *MSMEG_5817* gene was amplified from *M. smegmatis* gDNA using primers 5′-TGGCCAGCCGCCGTAGTGCCGAT-3′ and 5′-GGAATTCTACGGGTCGATACGCACCAC-3′ and cloned into the *Mlu*NI and *Eco*RI sites of pMV261 [29] with a gentamicin resistance cassette replacing kanamycin resistance conferred by the *aph* gene at the *Nsi*I sites. The derived plasmid, pMYC85c was transformed via electroporation into Myc85. Transformants were selected on 7H10 plates containing kanamycin and gentamicin and designated Myc85c.

### Macrophage infection assay

J774A.1 cells were suspended in complete RPMI and seeded into 24-well plates at 10^5^ cells/well, then incubated overnight at 37°C, 5% CO_2_. *M. smegmatis* WT, Myco132, Myc85 and Myc85c were passed through a 26-gauge needle ten times to disperse clumps [41] and then added to macrophage cells at an MOI of 10∶1. Phagocytosis was permitted during a 1 h incubation at 37°C, 5% CO_2_, after which macrophage cells were washed thoroughly with PBS to remove non-phagocytosed mycobacteria. RPMI containing 10 µg/ml gentamicin to kill extracellular bacteria, was added to the infected macrophage monolayer and incubated for a further 0, 4, 8, 24 and 48 h at 37°C, 5% CO_2_. At each time point macrophage cells were washed again with PBS and the intracellular mycobacteria released by lysing macrophage cells with ice-cold water. The recovered mycobacteria were serially diluted and plated onto BHI agar supplemented with the appropriate antibiotic to determine the colony forming units (CFU). The statistical significance of the data was evaluated by unpaired Student's *t* test.

### Luciferase assay

*M. smegmatis* WT and Myco132 were prepared from stationary phase cultures by washing in cold PBS and resuspending in 3.5% formaldehyde for 20 min, followed by additional washes in PBS. Serial dilutions for enumeration of CFU were performed prior to the addition of formaldehyde, to allow inoculation of equivalent cell numbers in the stimulation assays. Three biological replicates were prepared for each isolate. RAW264.7 mouse macrophage cells stably expressing the NF-κB-dependent ELAM-luciferase reporter construct [42] were a kind gift from Ashley Mansell, Monash Institute of Medical Research, Victoria, Australia. RAW264.7 cells were seeded at 2×10^4^ cells/well of a 96-well flat bottomed tissue culture plate in 100 µl LPS-free RPMI 1640 media (GIBCO) plus 0.5 mg/ml G418, supplemented with 10% FCS and 1 mM L-glutamine and incubated overnight at 37°C, 5% CO_2_. Cells were then stimulated with 10^5^ cfu mycobacteria in triplicate, per biological replicate for 3.5 h at 37°C, 5% CO_2_. After removing the media, 50 µl of 1× Promega passive lysis buffer was added to each well (5 min at room temperature). 20 µl from each well was transferred to a white opaque flat-bottomed 96 well plates, and 30 µl Luciferase assay reagent (Promega) added. Luciferase activity was measured with a Fluro-Optima luminometer. The statistical significance of the data was evaluated by paired Student's t test.

### *In vitro* growth and stress assays

To set up *in vitro* growth curves, a saturated 10 ml starter culture was diluted to OD_600_ = 0.5 in 1 ml BHI and used to inoculate 50 ml BHI. Broths were incubated at 39°C under constant orbital shaking and OD_600_ measured at various time points over a 48 h growth period. For acid stress assays, a 1 in 500 dilution of 10 ml starter culture was used to inoculate 50 ml BHI at standard pH (pH 7) and incubated at 39°C with shaking to an OD_600_ of ∼1.0 was reached. For pH stress experiments, cultures were pelleted by centrifugation at 2500× *g* for 5 min and washed once with BHI (pH 7, 5 or 3), and then resuspended in 10 ml BHI (pH 7, 5 or 3) at OD_600_ = 0.5. All broths were again incubated at 39°C with shaking and 1 ml removed for viable count enumeration after 0, 3 and 6 h. To assess the affect on growth of H_2_O_2_ stress, culture pellets were washed and resuspended in BHI (pH 5). H_2_O_2_ was added to each broth at a final concentration of 0, 2.5, 5, and 7.5 of 10 mM and cultured for 6 h at 39°C with shaking. At 0 and 6 h, 1 ml was removed to determine the viable count. In each case, results are expressed as a percentage of the CFU observed at 0 h.

### Quantification of phagocytosed mycobacteria by FACS

SYTO9 or SYTO62 fluorescent nucleic acid dye (Molecular Probes) was added to mycobacteria at a final concentration of 200 nM. Mycobacteria were vortexed briefly and incubated at room temperature for 30 min protected from light. Stained mycobacteria were collected by centrifugation at 3500× *g* for 5 min. Pellets were washed seven times with PBS and then resuspended in RPMI for macrophage infection. Macrophages were incubated with fluorescently stained mycobacteria (MOI 10∶1) at 37°C, 5% CO_2_ for 1 h. Non-phagocytosed bacteria were removed by washing the infected macrophage monolayer with PBS. Infected macrophages were harvested by treatment with trypsin/EDTA. Cells were centrifuged and the pellets resuspended in 4% formaldehyde solution. Samples (11,000–15,000 macrophage cells) were acquired and analysed using a Beckman Coulter FC500 flow cytometer and CXP software.

### RNA isolation and RT-PCR

For intracellular *M. smegmatis* samples, 10^8^ J774A.1 cells were seeded in 75 cm^2^ vented T-flasks containing 10 ml complete RPMI and incubated overnight at 37°C, 5% CO_2_. Macrophages were infected with *M. smegmatis* strains (MOI 10∶1) which had been passed through a 26G needle. Infected cells were incubated at 37°C, 5% CO_2_ for 1 h before non-phagocytosed bacteria were removed by washing with PBS. Complete RPMI was then replaced on the infected macrophage monolayer and incubated for a further 4 h. Extracellular samples were treated in the same manner, except macrophages were not present during the mock infection. Intracellular *M. smegmatis* were harvested from J774A.1 cells by differential lysis as described by Monahan et al [43]. Extracellular *M. smegmatis* were harvested by centrifugation at 2,500× *g* for 5 min and the pellet resuspended in 1 ml Qiagen RNA Protect Bacteria Reagent (Qiagen) and processed according to the manufacturer's instructions.

For total RNA isolation, 40 mg bacterial pellet was rinsed with 400 µl 0.5% Tween 80 and re-pelleted at 16,200× *g* for 1 min. The bacterial pellet was then resuspended in 720 µl lysis buffer (20 mM sodium acetate pH 4.4, 0.5% SDS, 1 mM EDTA pH 8.0, 100 µg/ml proteinase K) and transferred to a 2 ml screw-capped microfuge tube, containing 0.5 g of 425–600 microns acid-washed glass beads (Sigma) and 1 ml phenol/chloroform/isoamyl alcohol (125∶24∶1, pH 4.7). Bacterial cell lysis was achieved using a reciprocal shaker (Savant B10 101 Fast Prep) on speed setting 5.0 for three 30 s pulses. The aqueous phase was collected by centrifugation at 16,200× *g* for 10 min, removed to a new microfuge tube, and then re-extracted once with an equal volume of phenol (pH 4.3)/chloroform/isoamyl alcohol (25∶24∶1) and a final time with chloroform/isoamyl alcohol (24∶1). The RNA was precipitated with an equal volume of isopropanol, incubated for 30 min at −80°C and collected by centrifugation at 16,200× *g* for 30 min at 4°C. The pellet was washed with 70% ethanol, dried under vacuum and resuspended in 30 µl RNase-free water. Total RNA was treated with 2 units RQ1 DNase I (Promega) for 20 min at 37°C. The total RNA was then purified further using a Qiagen RNeasy purification column (Qiagen) according to the manufacturer's instructions.

First strand cDNA was synthesised from 5 µg total RNA and 500 ng random hexamer using Superscript III reverse transcriptase (Invirtogen) according to the manufacturer's instructions. PCR was then performed with 5 µl cDNA template in 1× reaction buffer, 1.5 mM MgCl_2_, 200 µM dNTPs, 10 µM forward and reverse primers (as described for Myco132 complementation) and 1.25 units *Taq* DNA polymerase. The PCR conditions were 95°C hot start for 7 min, followed by 94°C denaturation for 30 s, 55°C annealing for 30 s and 72°C extension for 1.5 min for 40 cycles and a final 72°C extension for 5 min. PCR products were separated on a 1% agarose gel containing 0.4 µg/ml ethidium bromide and visualised by UV transillumination.

### AGT enzyme assays

Enzyme extract was prepared as described by Sakuraba et al [40] with a few modifications. Briefly, *M. smegmatis* strains were grown in 500 ml BHI to an OD_600_ = 1.7–1.8. Cells were collected by centrifugation at 5000× *g* for 20 min and resuspended in 30 ml lysis buffer (20 mM potassium phosphate buffer [pH7.5], 20% glycerol, 1 mM EDTA, 0.1 mM DTT, 1 mM phenylmethylsulfonyl fluoride, 10 mg/ml lysozyme) and incubated on ice for 30 min. Samples were then sonicated 3×10 sec with a 30 sec pause on ice between each burst. Samples were centrifuged at 15000× *g* for 40 min and the supernatant used as enzyme extract for AGT assays. Total protein concentration of the enzyme assay was determined by Bradford assay [44] using BSA as the standard. AGT activity assay was adapted from two previously described methods [45] [40]. Enzyme extract containing 0.2 mg total protein was incubated at 37°C for 1 h with 20 mM alanine, 5 mM glyoxylate, 20 µM pyridoxal 5′-phosphate and 100 mM potassium phosphate buffer (pH 7.5) in a final volume of 0.8 ml. The reaction was stopped with the addition of 100 µl 50% trichloroacetate and centrifuged at 16,200× *g* for 10 min. The supernatant was removed and neutralised with the addition of 1.1 ml 0.27 M Tris-HCl. Pyruvate produced was then assayed using a Cary 100 Bio UV-visible spectrophotometer to measure the extinction of NADH in the presence of LDH. This was achieved by adding 0.4 ml AGT assay sample to a spectrophotometer cuvette with 0.23 M Tris-HCl (pH8.4), 0.12 mM NADH, 0.03% BSA and .075 units LDH in a final volume of 3 ml. Extinctions were measured at 340 nm over 6 min.
